# Optical Genome Mapping and Single Nucleotide Polymorphism Microarray: An Integrated Approach for Investigating Products of Conception

**DOI:** 10.3390/genes13040643

**Published:** 2022-04-03

**Authors:** Nikhil Shri Sahajpal, Ashis K. Mondal, Sudha Ananth, Chetan Pundkar, Kimya Jones, Colin Williams, Timothy Fee, Amanda Weissman, Giuseppe Tripodi, Eesha Oza, Larisa Gavrilova-Jordan, Nivin Omar, Alex R. Hastie, Barbara R. DuPont, Lawrence Layman, Alka Chaubey, Ravindra Kolhe

**Affiliations:** 1 Department of Pathology, Medical College of Georgia, Augusta University, Augusta, GA 30912, USA; nsahajpal@augusta.edu (N.S.S.); amondal@augusta.edu (A.K.M.); sananth@augusta.edu (S.A.); kjones2@augusta.edu (K.J.); colwilliams@augusta.edu (C.W.); eoza@augusta.edu (E.O.); nomar@augusta.edu (N.O.); 2 Department of Pathobiology, College of Veterinary Medicine, Auburn University, Auburn, AL 36849, USA; cyp0003@auburn.edu; 3 Cytogenetics Laboratory, Greenwood Genetic Center, Greenwood, SC 29646, USA; tfee@ggc.org (T.F.); dupont@ggc.org (B.R.D.); 4 Medical College of Georgia, Augusta University, Augusta, GA 30912, USA; a.weissman44@gmail.com (A.W.); gtripodi@augusta.edu (G.T.); 5 Department of Obstetrics and Gynecology, Medical College of Georgia, Augusta University, Augusta, GA 30912, USA; lgavrilovajordan@augusta.edu (L.G.-J.); lalayman@augusta.edu (L.L.); 6 Bionano Genomics Inc., San Diego, CA 92121, USA; ahastie@bionanogenomics.com (A.R.H.); achaubey@bionanogenomics.com (A.C.)

**Keywords:** optical genome mapping, microarray, products of conception

## Abstract

Conventional cytogenetic analysis of products of conception (POC) is of limited utility because of failed cultures, as well as microbial and maternal cell contamination (MCC). Optical genome mapping (OGM) is an emerging technology that has the potential to replace conventional cytogenetic methods. The use of OGM precludes the requirement for culturing (and related microbial contamination). However, a high percentage of MCC impedes a definitive diagnosis, which can be addressed by an additional pre-analytical quality control step that includes histological assessment of H&E stained slides from formalin-fixed paraffin embedded (FFPE) tissue with macro-dissection for chorionic villi to enrich fetal tissue component for single nucleotide polymorphism microarray (SNPM) analysis. To improve the diagnostic yield, an integrated workflow was devised that included MCC characterization of POC tissue, followed by OGM for MCC-negative cases or SNPM with histological assessment for MCC-positive cases. A result was obtained in 93% (29/31) of cases with a diagnostic yield of 45.1% (14/31) with the proposed workflow, compared to 9.6% (3/31) and 6.4% (2/31) with routine workflow, respectively. The integrated workflow with these technologies demonstrates the clinical utility and higher diagnostic yield in evaluating POC specimens.

## 1. Introduction

Clinically recognized pregnancy losses (at any gestational age) occur in ~15–25% of pregnancies and is a traumatic event for women and their families [1]. The rate of miscarriage (pregnancy loss < 20 weeks′ gestation) is reported to be ~15–20% [2], while 1/100 pregnancies result in stillbirth (pregnancy loss ≥ 20 weeks) in the US [3]. The majority of miscarriages are spontaneous and a significant number result from genetic anomalies influenced by maternal age [1,4,5]. The rate of miscarriage increases with age, as women of ≤35 years reported a 9–12% rate of miscarriage in 6-12 weeks [6,7]; >35 years of age had a high incidence of trisomic pregnancies [4], and those >40 years of age had an ~50% rate of miscarriage [1,7,8]. In contrast, recurrent pregnancy loss is a disorder defined as two or more failed clinical pregnancies. The standard screening protocol as recommended by the American Society for Reproductive Medicine (ASRM) comprises determining parental karyotypes, antiphospholipid antibodies, uterine cavity evaluation, thyrotropin, and prolactin levels after two consecutive failed clinical pregnancies. Chromosomal analysis of products of conception (POC) has been deemed useful in the setting of ongoing therapy for recurrent pregnancy loss (RPL) [9,10]. 

Chromosomal abnormalities provide a genetic diagnosis that is critical in understanding the cause of miscarriage and for recurrent-risk counseling that might help identify familial chromosomal rearrangements that predispose the parents to these events or the birth of children with genetic defects. Most pregnancy losses are marked with aneuploidies detected through traditional cytogenetic techniques [11,12]. However, the techniques are limited by the requirement of procuring live dividing cells, possible microbial or maternal cell contamination (MCC), and poor chromosome morphology that preclude a definitive diagnosis. The challenges associated with the cytogenetic analysis of POC demonstrate the need for alternate technologies or methodologies to improve the diagnostic yield in this important area of reproductive medicine.

Optical genome mapping (OGM) is an emerging technology that has demonstrated the potential to replace conventional cytogenetic methods in several recent clinical studies. The OGM technology is highly sensitive, with an ability to detect all classes of clinically significant genome-wide SVs (aneuploidies, CNVs, balanced genomic rearrangements, repeat contraction, repeat expansions, and mosaicism) [13,14,15]. The use of OGM technology is of significant advantage in the analysis of POC tissue, as it precludes the requirement for culturing and eliminates the risk of associated microbial contamination. However, MCC remains a challenge in a significant number of cases and impedes a definitive diagnosis. Although microarrays have been implemented as a reliable method of genome-wide analysis for pregnancy losses, and have shown increased diagnostic yield over conventional karyotyping [16,17,18,19,20], they cannot circumvent a high percentage of MCC contamination. To address the significant variations in maternal and fetal content in the POC specimens, an additional pre-analytical step was added where the specimens were subjected to hematoxylin and eosin (H&E) histologic staining and examination by a pathologist to assess the presence of chorionic villi before any diagnostic test was performed on these specimens. The slides were further macro-dissected for the enrichment of chorionic villi and then analyzed using SNP microarray methodology. Thus, in this brief report, we propose an integrated workflow that includes MCC characterization of POC tissue, followed by OGM for MCC-negative cases or single nucleotide polymorphism microarray (SNPM) with histological assessment for MCC-positive cases to improve the diagnostic yield in cytogenetic analysis of POC tissue.

## 2. Materials and Methods

### 2.1. Patient Specimen and Study Design

**Figure 1:**
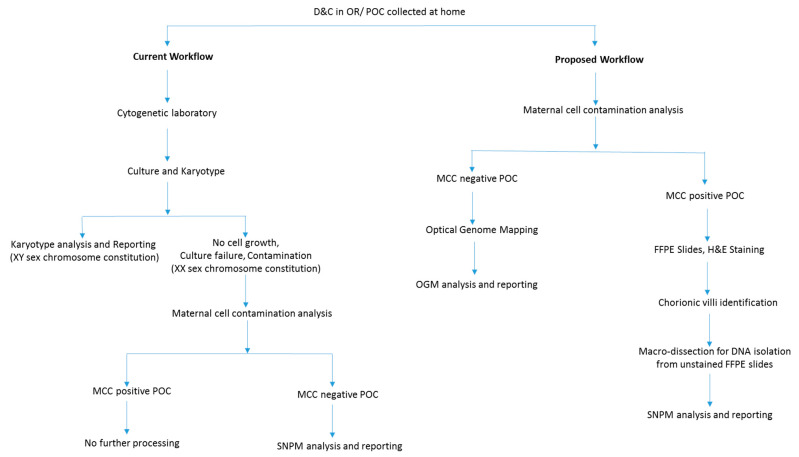
Flowchart depicting a modified diagnostic laboratory workflow compared to routine testing for high diagnostic yield in evaluating POC.

From 1^st^ January till 30th June 2021, nine POC cases were prospectively collected and analyzed using the proposed workflow along with conventional clinical testing. Additionally, we re-evaluated 22 cases (for whom the formalin-fixed paraffin embedded (FFPE) blocks were available) on which no clinical information (failed culture and high MCC precluding analysis with karyotyping and SNPM, respectively) could be achieved with conventional cytogenetic analysis. Thus, the present study evaluated 31 POC specimens that included, 9 prospectively collected cases and 22 retrospective cases. The study was designed so that the 9 prospective cases were evaluated with the proposed workflow alongside current workflow (Figure 1). The 22 retrospective cases that failed to yield result with current workflow because of failed cultures and MCC were analyzed with OncoScan microarray after histological assessment as per the proposed workflow. Clinical information was obtained from patient charts under an IRB-approved protocol, and archival FFPE blocks with slides were retrieved for review. The study was approved by the IRB A- BIOMEDICAL I (IRB REGISTRATION #00000150), Augusta University. HAC IRB # 611298. Based on the IRB approval, the need for consent was waived; all protected health information (PHI) was removed, and all data were anonymized before their accessing for the study.

### 2.2. Maternal Cell Contamination

DNA samples from the mother′s blood and POC (fetal sample) were processed for maternal cell contamination analysis. Briefly, both samples were subjected to polymerase chain reaction (PCR) amplification of nine polymorphic STR loci plus amelogenin. Amplicons from the maternal and fetal samples were sized by capillary electrophoresis, and then directly compared to determine if maternal cell contamination was present in the fetal sample.

### 2.3. Optical Genome Mapping

For POC negative for MCC, ~15 mg of POC section was used to isolate ultra-high molecular weight (UHMW) molecules using the Bionano Prep SP DNA Isolation Kit. Subsequently, the Bionano Prep DLS Labeling Kit was used to fluorescently label long molecules at specific sequence motifs throughout the genome. The labeled DNA was loaded onto Saphyr chips for linearization and imaging in massively parallel nanochannel arrays. The observed unique patterns on single long DNA molecules were used for de novo genome assembly and structural variant calling via the Bionano Solve pipeline (version 3.6). 

### 2.4. Histological Assessment and SNPM Analysis

**Figure 2:**
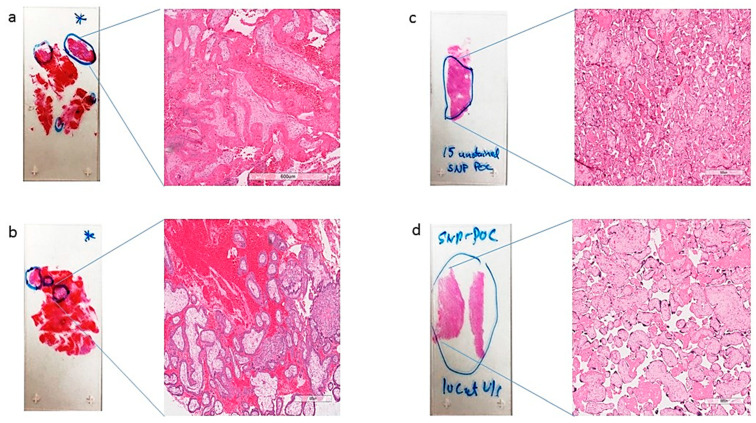
Representative H&E slides (**a–d**) identifying (fetal tissue) marked in blue circles, with a zoomed-in view at 600 µm magnification, those marked for macrodissection and DNA isolation by a surgical pathologist.

**Figure 3:**
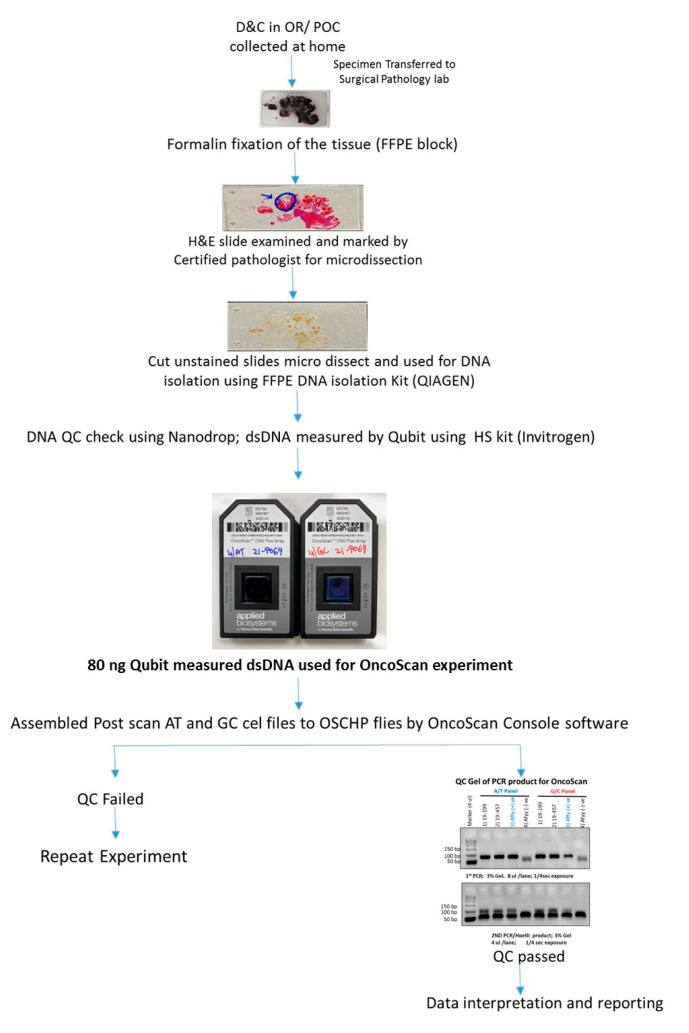
Flowchart depicting the detailed workflow for MCC-positive POC tissue with histological assessment and OncoScan microarray analysis and reporting.

FFPE slides were stained with H&E and examined by a board-certified pathologist (RK) to identify chorionic villi (fetal tissue) and marked for macrodissection (Figure 2 and Figure 3). Following fetal tissue enrichment using macrodissection, DNA was isolated using a QIAamp DNA FFPE tissue kit (Qiagen, Hilden, Germany). The isolated DNA was analyzed using the whole-genome SNP microarray following the manufacturer′s protocol (OncoScan^®^ FFPE assay kit, ThermoFisher Scientific, Waltham, MA, USA). The platform consists of 220,000 markers throughout the entire genome. The test compares the samples to control samples from the HAPMap set of 270 individuals. The raw data was analyzed using the Chromosome Analysis Suite (ChAS) 4.0 software and were matched to in silico FFPE reference sets.

### 2.5. Data Analysis

The analysis results were classified and reported as follows: gains and losses of an entire chromosome as an aneuploidy, gains, and losses (>10 MB) of regions of a chromosome as partial aneuploidy, and terminal loss from one chromosome coupled with a terminal gain from another chromosome as an unbalanced translocation. The following databases were used to assess the clinical significance of genomic aberrations less than 5 Mb: the database of genomic variants (DGV), DatabasE of genomiC varIation, and Phenotype in Humans using Ensembl Resources (DECIPHER), Online Mendelian Inheritance in Man (OMIM), and PubMed. The American College of Medical Genetics and Genomics (ACMG) microarray reporting guidelines were used to classify the variants as pathogenic, likely pathogenic, and variants of uncertain clinical significance. Notably, benign and likely benign variants were not reported. 

## 3. Results

### 3.1. Patient Characteristics 

In the present study, 31 POC samples were evaluated, of which 28 did not yield clinical diagnostic results with conventional cytogenetics. The mean age of women was 33.5 ± 5.1 (range 22–44 years), with 22 cases of spontaneous miscarriage and 9 of RPL. Women with RPL had a total of 34 losses. Sixteen women had a history of no prior live births, while eight, four, and three women had one, two, and three prior births, respectively. Of these cases, twenty-seven women had a miscarriage in <20 weeks of gestation, three had intra-uterine fetal demise, and one had a stillbirth (Table 1).

### 3.2. Prospective Sample Processing with Current and Proposed Workflow

In the current workflow, of the nine prospectively collected POC cases, only two samples could be cultured successfully and were analyzed with karyotyping. Of the seven remaining cases, six were identified with MCC contamination (>50%), and were not processed further. One sample that was negative for MCC was processed with SNPM analysis. 

In the proposed workflow, of the nine samples, six were identified with MCC contamination and were subjected to histological assessment and SNPM analysis, while three samples were analyzed with OGM.

### 3.3. Optical Genome Mapping Analysis

**Figure 4:**
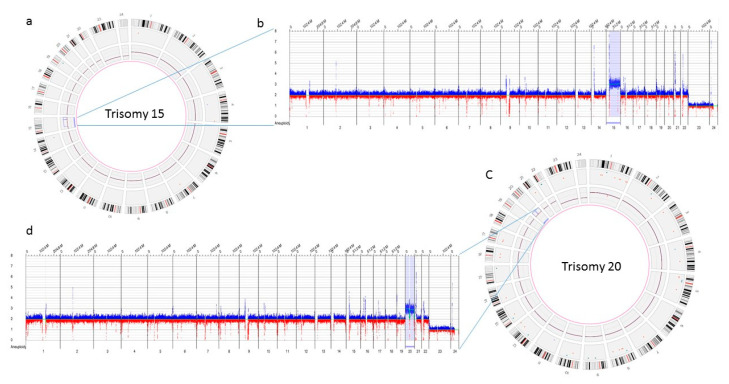
Optical genome mapping identifying trisomy 15 (**a**) circos plot showing trisomy 15 (**b**) copy number track view with trisomy 15, (**c**) circos plot showing trisomy 20 (**d)** copy number track view with trisomy 20.

The three samples analyzed using OGM achieved excellent quality control performance metrics with an average N50 of 292 kbp (recommended >220 kbp), map rate of 87% (recommended >70), and label density of 15.8 (recommended 15–17). OGM was concordant in identifying the results observed with conventional cytogenetic analysis. OGM confirmed trisomy 15, and 20 in the first two cases, previously identified by karyotyping, while in the third case no reportable SVs or CNVs were detected using OGM, as previously reported with microarray technology (Figure 4). 

### 3.4. Histological Assessment and SNPM Analysis

Of the twenty-eight FFPE POC samples macro-dissected for chorionic villi and analyzed utilizing the OncoScan assay, results were obtained in 92.8% (26/28) of cases, where traditional cytogenetic analysis had not yielded a definitive result. Maternal cell contamination was identified in 7.1% (2/28) cases, wherein copy number changes could not be identified; however, both cases were identified as being males. Genetic aberrations were detected in 42.8% (12/28) of cases, while no reportable copy number aberrations or absence of heterozygosity (AOH) were detected in 57.1% (16/28) cases. (Table 2).

### 3.5. Proposed Workflow Compared to Conventional Cytogenetic Testing

In the proposed workflow, a result was obtained in 93% (29/31) of the cases (26 with microarray and 3 with OGM) when compared to 9.6% (3/31) with routine workflow, while the diagnostic yield was 45.1% (14/31) with the proposed workflow when compared to 6.4% (2/31) with conventional cytogenetic testing.

### 3.6. Selected, Interesting Clinical Cases

**Figure 5:**
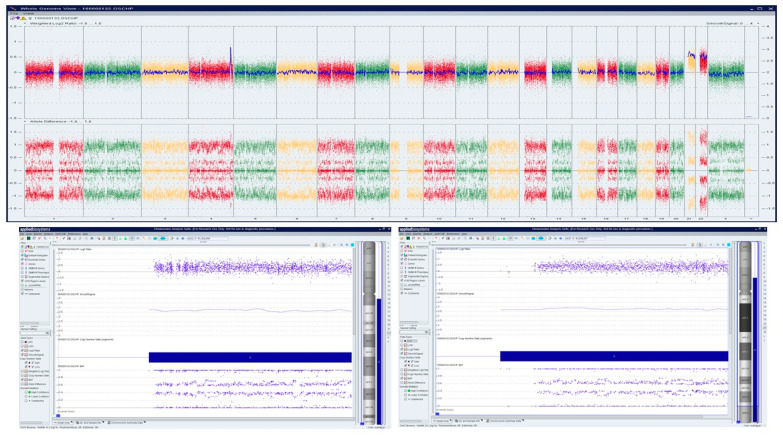
OncoScan analysis identifying double mosaic trisomies involving chromosomes 21 and 22 (ascertained as ~70% mosaic gain), and a copy number gain of Chr 4q, a variant of uncertain significance: Upper panel shows whole-genome view, lower panel shows log2ratio, smooth signal, and B-Allele Frequency (BAF) of chr 21, c) log2ratio, smooth signal, and BAF of chr 22.

A Caucasian female (<40 years) with a history of over three miscarriages and multiple failed attempts at karyotyping had a miscarriage in the 1st trimester in her subsequent pregnancy. The patient had a medical history of Hashimoto′s thyroiditis, asthma, obesity, insulin resistance, Cushing’s disease, and depression. H&E stained slides of FFPE tissue were marked by a surgical pathologist for chorionic villi, which were macro-dissected for DNA isolation. OncoScan analysis identified double mosaic trisomies involving chromosomes 21 and 22 (ascertained as ~70% mosaic gain), and a copy number gain of Chr 4q, a variant of uncertain significance (Figure 5).

**Figure 6:**
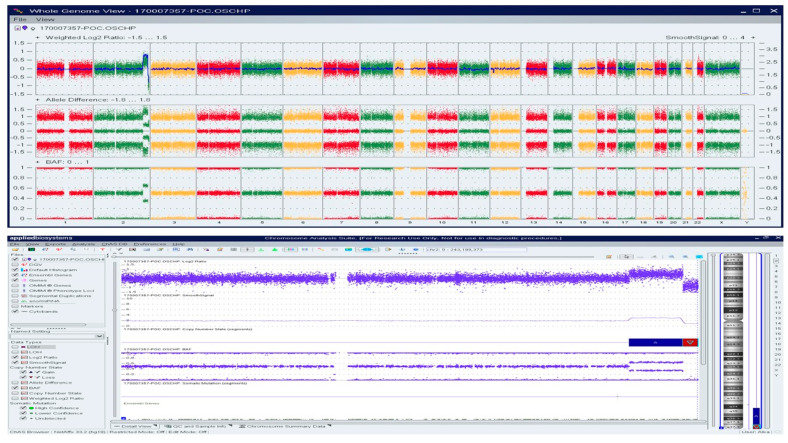
OncoScan analysis identified 22.6 Mb copy gain of Chr 2q34q37.2 and a 6.4 Mb copy loss of Chr 2q37.2q37.3, indicating a complex rearrangement involving the long arm of chromosome 2: Upper panel shows the whole-genome view.Lower panel shows the log2ratio, smooth signal, and BAF of chr 2.

A Caucasian female (>30 years) with a history of over three miscarriages and multiple failed attempts at karyotyping had a miscarriage in the second trimester of her subsequent pregnancy. The patient had a medical history of hypothyroidism, recurrent pregnancy loss, and was reported to harbor a compound heterozygous *MTHFR* gene mutation. Cytogenetic/karyotyping could not be performed on this specimen, as no metaphase cells were present. On analyzing the FFPE POC tissue with macrodissection, a 22.6 Mb copy gain of Chr 2q34q37.2 and a 6.4 Mb copy loss of Chr 2q37.2q37.3 was identified, indicating a complex rearrangement involving the long arm of chromosome 2 (Figure 6).

## 4. Discussion

Pregnancy losses occur in ~15–25% of clinically recognized pregnancies, with approximately 50% caused by chromosomal abnormalities. Identification of these genetic aberrations is essential in understanding the underlying cause of these painful and traumatic events. Defining the genetic etiology of these pregnancy losses allows these patients to understand the reason for their miscarriage, bringing some degree of closure, and may also predict the recurrence risk for the parents [1,4,5]. The chromosomal abnormalities in the POC tissue can also suggest further testing protocols, with certain abnormalities such as aneuploidies that have not been associated with recurrence risk and may not require further testing, while unbalanced chromosomal translocation or inversion and euploid POC are suggestive indications for further testing, including a whole-genome chromosomal analysis of parents, and RPL workup, respectively [21]. However, performing chromosome analysis on POC tissue is not always feasible and a diagnosis is precluded because of a variety of reasons. Conventional cytogenetic analysis is limited because of the requirement of live cells for culturing, culture contamination, and maternal cell contamination. Further, conventional techniques are limited in resolution or biased to certain loci and thus, several genetic events may remain cryptic. The low diagnostic yield observed with conventional cytogenetic technique/workflow led us to devise an integrated workflow that bypasses the requirement for culturing (a major time-consuming step). The proposed workflow begins with MCC characterization of POC tissue, followed by OGM for MCC-negative cases or SNPM with histological assessment for MCC-positive cases.

Optical genome mapping is an emerging cytogenetic technology that can detect all classes of SVs and CNVs in a single assay as compared to conventional cytogenetic techniques (karyotyping, FISH, and SNPM). Although only three samples were analyzed using OGM, the quality control metric demonstrates the feasibility of using OGM for the analysis of POC tissue. Several of the POC tissues remain uncharacterized because of failed cultures that can be adequately addressed by OGM technology, as UHMW DNA was isolated directly from the POC tissue. Further, the use of OGM reduces the time to reach a diagnosis, as cultures may take weeks before sufficient cells are available for analysis. As OGM technology has demonstrated the ability to identify additional/novel structural variants that were missed by conventional methods in several phenotypes [13,14,15], it would be interesting to see if OGM would detect additional aberrations in otherwise normal POC genomes evaluated with conventional techniques (Supplementary File S1). 

For the MCC-positive cases, the addition of a pre-analytical step with histological assessment and SNPM analysis led to a definitive result in 90.5% (19/21) of samples. The two samples that still showed maternal cell contamination were identified as males, although a copy number assessment could not be made. It is important to note that maternal cell contamination as low as 5–10% is detectable with SNP microarray, and insignificant levels of MCC have been reported in 93% of fresh and 60% of FFPE tissue. However, an MCC of less than 50% may not severely affect the detection of segmental or whole chromosome imbalances [18]. The high diagnostic yield in this study is partially attributed to the histological assessment of the FFPE POC tissue for chorionic villi, which seems to be an ideal methodology upstream of SNP microarray analysis. The histological assessment helps rule out the presence of maternal tissue and enables macrodissection of chorionic villi, to enrich the fetal tissue for DNA isolation and SNP microarray analysis. The major limitation that precludes a definitive clinical diagnosis with SNP microarrays is the issue of MCC, which can be addressed with this approach in the pre-analytical stage of the assay. The ability of the platform to yield a diagnosis with minimal MCC should therefore be more fully exploited for clinical insights, and the histological assessment of the FFPE POC tissue might be a necessary additional step that needs to be incorporated in the clinical workup for improved diagnosis. The higher performance and the clinical utility of the SNP microarray were further highlighted by the two RPL cases where multiple attempts were made to obtain a karyotype analysis, but no clinical diagnosis could be achieved, and a definitive clinical diagnosis with genetic aberrations was detected on analyzing the FFPE POC tissue. 

Overall, we propose an integrated workflow with these technologies that demonstrated the clinical utility and higher diagnostic yield in evaluating POC specimens in this study. The OGM technology is highly sensitive to detect several classes of structural variants and copy number variants, while SNPM with the addition of this pre-analytical step has a significant potential to improve clinical diagnosis in this important area of reproductive medicine. Although, the study is limited to a small sample size, the critical insights obtained with this distinct approach address the key issues associated with current diagnostic testing, and might serve as an important factor to improve diagnostic yield in POC analysis.

## 5. Limitations of the Study

The present study has a few limitations that include the small samples size. Further studies are needed to evaluate the proposed approach and assess the utility of each arm of the proposal (OGM and SNPM with histological assessment). Additionally, OGM could not be performed on FFPE tissue with histological assessment as the technology in its current iteration is not compatible with fragmented DNA. As a result, only a subset of samples with no MCC could be examined with OGM technology, and additional studies with higher sample numbers are needed to evaluate the clinical utility of OGM in POC analysis. 

## Figures and Tables

**Table 1:** Clinical characteristics.

Patient Characteristic	Classification	No.
Anamnestic data		
Age (Mean ± SD)		33.4 ± 5.1
No. of previous losses	2	4
	3	4
	4	0
	5	1
	6	1
	7	1
No. of previous live births	0	16
	1	8
	2	4
	3	3
Miscarriage	<20 weeks	27
	Intra-uterine fetal demise	3
	Stillbirths	1
Current Material		
Sporadic miscarriage		22
Recurrent pregnancy loss		9

**Table 2:** Abnormal genetic aberrations detected with SNP microarray and optical genome mapping on the product of conception.

S.No.	Trimester	ISCN Nomenclature	Size
OncoScan Analysis			
1	9 weeks, 1st	arr[hg19] (22) × 3	Entire chromosome 22
2	10 weeks, 1st	arr[hg19] 4q34.3(178,112,003-182,153,124)×3, (21,22) × 2~3	4.0 Mb Gain of chr 4 and Gain of entire chromosomes 21 and 22
3	8 weeks, 1st	arr[hg19] (8) × 2~3	Entire chromosome 8
4	9 weeks, 1st	arr[hg19] (8) × 3	146.1Mb
5	10 weeks, 1st	arr[hg19] (14) × 2~3	Entire chromosome 14
6	10 weeks, 1st	arr[hg19] (X) × 1~2	Entire chromosome X
7	14 weeks, 2nd	arr[hg19]2q34q37.2(214027641-236628038) ×3, 2q37.2q37.3(236228142-243052331) × 1	22.6Mb gain and 6.4Mb loss
8	11 weeks, 1st	arr [hg19] (1-21,X) × 2, (22)×2~3	Entire chromosome 22
9	8 weeks 5 days, 1st	arr[hg19] (15) × 2~3	Entire chromosome 15
10	6 weeks, 6 days, 1st	arr[hg19] (18) × 3	Entire chromosome 18
11	26 weeks 1 day, 2nd	arr[hg19] 2p21(43411752-44710936) × 3, 21q21.1(18762223-19136546) ×1	1.29 Mb Gain and 374 Kb Loss
12	Less than 3 weeks, 1st	arr[hg19] 13q14.11(42311546_42413745) × 1	102 Kb Loss
Optical Genome Mapping			
13	6 weeks, 1st	ogm[GrCh38](15) × 3	Entire chromosome 15
14	8 weeks, 1st	ogm[GrCh38](20) × 3	Entire chromosome 20

## Data Availability

All relevant data is provided in the manuscript. Please contact at rkolhe@augusta.edu for any raw data files and further analysis.

## Supplementary Materials

The following are available online at https://www.mdpi.com/article/10.3390/genes13040643/s1, Supplementary File S1: comparison of different technologies with OGM for variant classes.
